# Molecular diagnostic testing of diffuse gliomas in the real-life setting: A practical approach 

**DOI:** 10.5414/NP301110

**Published:** 2018-06-20

**Authors:** Michał Bieńkowski, Adelheid Wöhrer, Patrizia Moser, Melitta Kitzwögerer, Gerda Ricken, Thomas Ströbel, Johannes A. Hainfellner

**Affiliations:** 1 Institute of Neurology, Medical University of Vienna, Austria; 2 Department of Molecular Pathology and Neuropathology, Medical University of Lodz, Poland,; 3 Section of Pathology, Tirol-Kliniken Innsbruck, and; 4 Department of Pathology, University Hospital of St. Poelten, Karl Landsteiner University of Health Sciences, St. Poelten, Austria

**Keywords:** diffuse gliomas, IDH mutations, 1p19q co-deletion, TERT promoter mutations, MLPA

## Abstract

Typing of diffuse gliomas according to the WHO 2016 Classification of Tumors of the Central Nervous System is based on the integration of histology with molecular biomarkers. However, the choice of appropriate methods for molecular analysis and criteria for interpretation of test results is left to each diagnostic laboratory. In the present study, we tested the applicability of combined immunohistochemistry, direct sequencing, and multiplex ligation-dependent probe amplification (MLPA) for diagnostic assessment of *IDH1/2* mutation status, chromosome 1p/19q status, and *TERT* promoter mutations. To this end, we analyzed a consecutive series of 165 patients with diffuse low- and high-grade gliomas (WHO grade II and III) from three Austrian centers in which tissue specimens were routinely processed. We could reliably detect *IDH1/2* mutations by combining immunohistochemistry, direct sequencing, and MLPA analysis. MLPA analysis also allowed reliable detection of combined whole chromosomal arm 1p/19q codeletion when using carefully selected criteria providing an optimal balance between sensitivity and specificity. Direct sequencing proved to be suitable for identification of *TERT* promoter mutations, although its analytical performance remains to be assessed. To conclude, we propose a practicable combination of methods and criteria which allow reliable molecular diagnostic testing of diffuse gliomas in the real-life setting.

## Introduction 

Histopathology has been the main basis for brain tumor typing and classification and oncological treatment decisions for decades. Recent years have witnessed an enormous advance in the understanding of brain tumor genetics, which recently resulted in the integration of molecular markers in the WHO Classification of Brain Tumors [1]. Further, targeted antineoplastic therapies have been developed, paving the way to precision medicine also in neuro-oncology [2]. An ever increasing number of genetic brain tumor biomarkers is being discovered serving diagnostic, prognostic as well as predictive purposes [3, 4, 5]. It is important to note that the reliable clinical implementation of biomarkers requires validation of their analytical and clinical performance in adequately powered studies [6, 7]. 

Diffusely infiltrating gliomas are, per definition, a group of primary CNS tumors with astrocytic and/or oligodendroglial phenotype and diffuse infiltration of the surrounding brain tissue. The distinction of diffuse astrocytoma and oligodendroglioma on basis of histological phenotype is fraught with limited interobserver agreement, which has led to the opinion that classification of these tumors should be based on genetic features. Some leading experts in the field even argue that IDH mutation status and 1p 19q co-deletion status should overrule histological phenotype in tumor classification [8]. Further, two research groups have suggested independently that clinically meaningful prognostic classification of diffuse gliomas can be done on basis of three molecular markers, *i.e., IDH1/2* mutation, *TERT* promoter mutation, and 1p19q co-deletion, without the need of histological typing and grading [9, 10]. However, the recently published update of the WHO Brain Tumor Classification clearly states that for the time being histology remains indispensable for brain tumor typing, and rather needs to be integrated with molecular biology [1]. With regard to the applied methodology, it is left open which methods and criteria are used for molecular testing in the respective laboratory. Concerns, however, remain with regard to interlaboratory comparability and robustness of molecular test results [11]. This problem may be less eminent in case of IDH mutation, because the most frequent IDH mutation (R132H) can be immunohistochemically visualized by means of a mutation-specific antibody, which has been shown to yield reliable and reproducible test results [12]. Sequencing needs to be applied for identification of other *IDH* mutations and for analysis of *TERT* promoter sequence [12]. On the other hand, there are various methods which may be employed and are in use for copy number analysis of chromosome 1p/19q status, encompassing techniques at both cell and DNA level. The former include fluorescent in situ hybridization (FISH) and chromogenic in situ hybridization (CISH), while the latter multiplex ligation-dependent probe amplification (MLPA), PCR-based loss of heterozygosity (LOH) analysis and microarray-based comparative genomic hybridization (aCGH) [13]. Systematic validation in the framework of clinical trials has been performed only for FISH [14]. This technique is, however, a time-consuming method, and hybridization is restricted to small chromosomal regions on 1p and 19q, and thus does not directly prove whole-arm chromosomal losses. However, isolated segmental deletions within 1p36 (the region where the FISH 1p probe is located) have been described as relatively common in gliomas [15]. 

The aim of the present study was to assess the applicability of combined immunohistochemistry, direct sequencing, and MLPA analysis for molecular diagnostic testing of IDH, 1p/19q, and TERT status in a real-life setting. We focused on analyzing the practical aspects, identifying problems, and looking for solutions, and to provide recommendations for use in the daily diagnostic setting. 

## Materials and methods 

### Patients

The study group comprised a consecutive series of 165 adult patients with diffusely infiltrating gliomas (grade II and III) who underwent operation at the Departments of Neurosurgery at the University Hospitals Innsbruck, St. Pölten, and Vienna. All biopsy specimens were diagnosed between January 2013 and March 2015 at the Institute of Neurology, Medical University of Vienna. Histopathological diagnosis was based at that time on the criteria of the WHO 2007 Classification of Tumors of the Central Nervous System [16]. 

### Immunohistochemistry

Formalin-fixed paraffin-embedded tissue sections of 3 µm were used for immunohistochemistry. 

Immunohistochemical staining of EGFR and TP53 was performed on an automated staining platform (Autostainer Link 48, Dako/Agilent Technologies, Santa Clara, CA, USA), using EnVision FLEX+ (#K8002, Dako/Agilent Technologies) as a visualization system according to manufacturer’s recommendations with diaminobenzidine (DAB) as chromogen. Briefly, heat-induced epitope retrieval was performed using FLEX TRS high solution at 95 °C for 20 minutes. Primary antibody EGFR (#NCL-EGFR, clone EGFR.113, Novocastra, Leica Biosystems, Newcastle, UK) was used at a dilution of 1 : 50 for 120 minutes at room temperature. Primary antibody p53 (#M7001, clone DO-7, Dako/Agilent Technologies) was used at a dilution of 1 : 50 for 30 minutes at room temperature. 

Immunohistochemical staining of ATRX and IDH1R132H was performed manually. Briefly, endogen peroxidase was blocked using 0,9% H_2_O_2_ (in methanol) for 10 minutes. Heat-induced epitope retrieval was performed with 10 mM citrate buffer (pH6) in a steamer for 60 minutes. ATRX manual staining was performed using Shandon coverplates and Sequenza immunostaining racks (Thermo Fisher Scientific, Waltham, MA, USA). Primary antibody ATRX (#HPA001906, rabbit polyclonal, Sigma-Aldrich, St. Louis, MO, USA) was used at a dilution of 1 : 300 overnight at +4 °C. Then, REAL EnVision detection system (#K5007, Dako/Agilent Technologies) was used for visualization according to manufacturer’s recommendations with DAB as chromogen. IDH1 immunostaining was performed manually in a humidified chamber. Primary antibody IDH1R132H (#DIA H09L, clone H09, Dianova, Hamburg, Germany) was used at a dilution of 1 : 30 for 60 minutes at room temperature. For manual visualization EnVision FLEX+ (#K8002, Dako/Agilent Technologies) was used with DAB as chromogen. Finally, all sections were counterstained with Mayer’s hemalum solution. 

Of note, we have recently optimized our ATRX and IDH1^R132H^ immunostaining procedures, which are now performed on the automated staining platform Ventana Benchmark Ultra (Roche, Basel, Switzerland), using UltraView Universal DAB Detection Kit (#760-500, Ventana/Roche, Basel, Switzerland) as a detection system according to manufacturer’s recommendations. For ATRX staining, heat-induced epitope retrieval is performed for 64 minutes with Ultra CC1 solution. For IDH1R132H staining, heat-induced epitope retrieval is performed for 68 minutes with Ultra CC2 solution. Primary antibody ATRX (#HPA001906, rabbit polyclonal, Sigma-Aldrich) is used at a dilution of 1 : 300 for 60 minutes at 37 °C. Primary antibody IDH1R132H (#DIA H09L, clone H09, Dianova) is used at a dilution of 1 : 200 for 60 minutes at 37 °C. Primary antibody incubation is followed by an amplification step with Amplification Kit (#760-080, Ventana/Roche) used according to manufacturer’s recommendations. Counterstaining is performed with hematoxylin (#760-2021, Ventana/Roche) and bluing reagent (#760-2037, Ventana/Roche) used according to manufacturer’s recommendations. 

### DNA analyses

DNA was isolated from ten 5 µm FFPE-tissue sections using the QIAmp FFPE isolation kit according to the manufacturer’s instructions. The relevant *TERT*-promoter sequence as well as *IDH1* codon 132 and *IDH2* codon 172 were amplified from genomic DNA using the PCR conditions as shown in Table 1. All primer pairs were designed with alternate forward MTR and reverse M13 tails to facilitate primer sequencing of both DNA strands (primer sequences are shown in Table 1). Each PCR product was individually analyzed by direct sequencing on a 3130 DNA sequencer using the Big-Dye3.1 sequencing chemistry (Applied Biosystems, Carlsbad, CA, USA). In order to enhance sensitivity for the detection of the *IDH1* mutation in some cases we performed a nested PCR reaction, in which the reaction with IDH1/Ex4P primers was followed by a reaction with IDH1/Ex4 primers. *IDH1/2* sequencing was performed in all samples with negative staining and in 12 random samples with positive staining for validation. In order to detect *TERT* promoter mutations, sequencing was performed in all samples. The samples were considered mutated if the mutated peak height was equal to at least 25% of the wild-type peak. 

MLPA was performed using the SALSA MLPA kit and P088 probe mix (MRC Holland, Amsterdam, the Netherlands) according to manufacturer’s protocol. Samples were analyzed on a 3130 DNA sequencer (Applied Biosystems). In order to assess the reproducibility of this assay we randomly selected 38 cases which were subsequently reanalyzed: In 23 cases we repeated the MLPA analyses on the same DNA isolation, and in 15 cases we isolated a new batch of DNA from the same FFPE tissue and performed the MLPA analysis. In 9 of these 15 cases, the MLPA analysis was performed twice. 

### Statistics

Statistical analysis was performed using R version 3.4.3 [17]. χ^2^-test and Fisher’s exact test (depending on number of observations) were used to analyze the frequency of the analyzed markers in given subgroups. Spearman’s rho was used to analyze the correlation of values for each loci. Cohen’s weighted kappa statistics (calculated with psych package [18]) was applied to analyze the reproducibility of MLPA for 1p/19q co-deletion. Figures were prepared using the graphical packages: ggplot2, gridExtra, colorscape, and heatmap3 [19, 20, 21, 22]. 

## Results 

### Cohort summary

The patients were between 18 and 80 years old (mean 45.8 years, median 44 years). There were 84 males and 81 females. The patients were histopathologically typed according to the WHO Brain tumour Classification 2007 as astrocytic tumors in 80 cases, oligodendroglial tumors in 31 cases and as mixed gliomas in 54 cases (details are summarized in Table 2). 

### TP53, EGFR, and ATRX expression

Strong nuclear TP53 expression in the majority of tumor cells was observed in 77/164 cases (47%) and EGFR expression in 98/161 cases (61%). Loss of ATRX expression was detected in 52/162 cases (32%). Detailed distribution of all markers between histological diagnoses is presented in Table 3. 

### TERT promoter mutations

*TERT* promoter was sequenced in 158 cases, and the mutation was identified in 96/157 cases (61%). Detailed distribution of all markers between histological diagnoses is presented in Table 3. 

### IDH mutations

IDH1^R132H^ expression was observed in 91/165 cases (55%). MLPA analysis was done in 150 cases; wild-type status was noted in 68 cases (45.3%), *IDH1* *^R132H^* mutation in 80 (53.3%), *IDH1* *^R132C^* in 2 (1.3%), and *IDH2* ^R172K^ mutation 1 case (0.7%) (data not shown). Both staining and MLPA were available in 149 cases, 147 of which showed concordant results (kappa = 0.97); in 2 cases a small fraction of IDH1^R132H^-positive cells visible by immunostaining was not detected by MLPA. Upon sequencing of cases with negative IDH1^R132H^ staining, other mutations were detected in 5 cases (2× R172K, 1× R132C und 2× R132G). One of the *IDH2* *^R172K^* mutations was recognized as wild-type according to MLPA, while the *IDH1* *^R132G^* probe is not included in the MLPA panel. In 10 cases with positive IDH1^R132H^ staining, sequencing initially failed to detect the mutation. However, in 8/10 cases sequencing of the nested PCR product allowed to detect the mutation, while the remaining 2 cases were the same that were negative according to MLPA (likely due to too low proportion of the mutated allele). Overall, the concordance of sequencing with immunohistochemistry and MLPA was good (κ = 0.92 in both cases). 

### 1p/19q co-deletion detection

**Figure 1.**
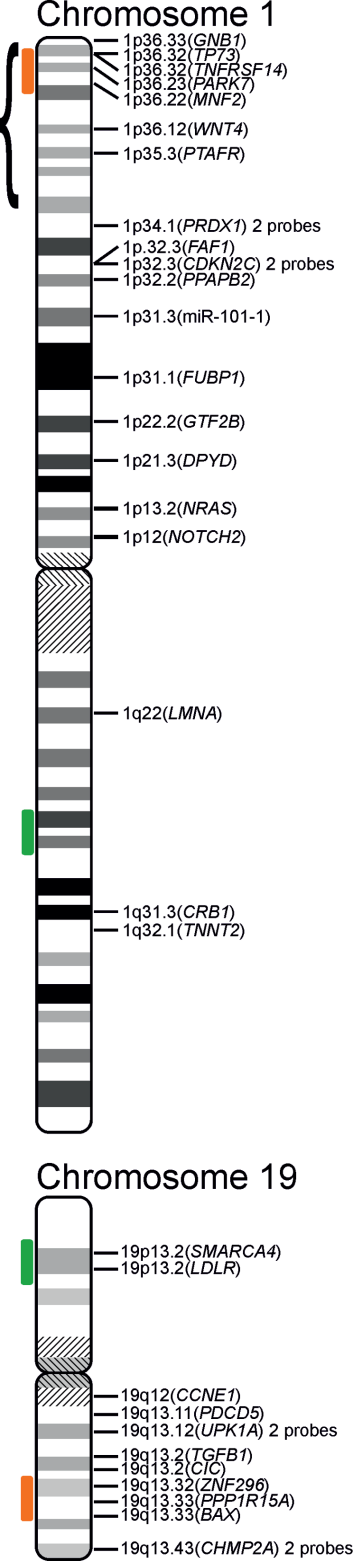
Location of MLPA probes for loci on chromosomes 1 and 19 (target genes are specified in parentheses). Orange and green bars mark location of the most commonly used FISH probes. The brace marks the region with recurrent partial deletions in gliomas (1p34.2-1pter) [26].

**Figure 2.**
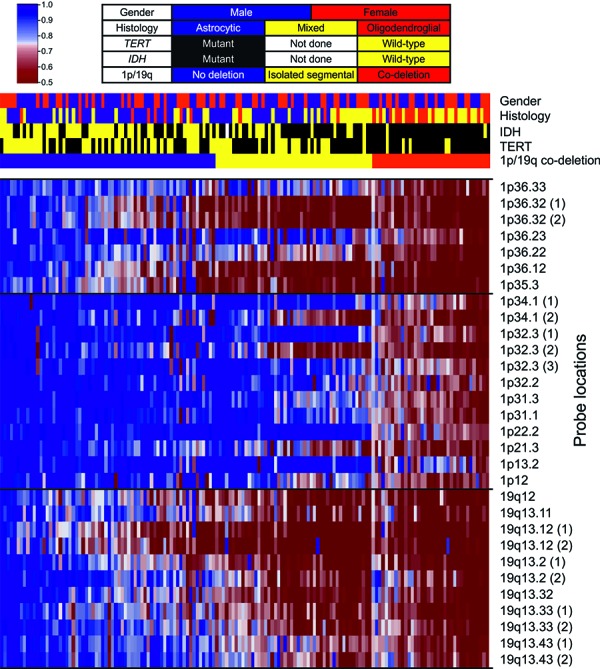
Heat map showing the relative copy number for each probe. Probes are ordered according to chromosomal location (the horizontal lines separate the commonly deleted, subtelomeric part of 1p, the rest of 1p and 19q). Samples are ordered according to the average value for the 30 analyzed loci. Color scale was limited to 0.5 – 1 (values beyond this range are presented as the extreme). Top bars show patient gender, tumor histology as well as IDH, *TERT*, and 1p/19q status (color codes presented in the Table above).

**Figure 3.**
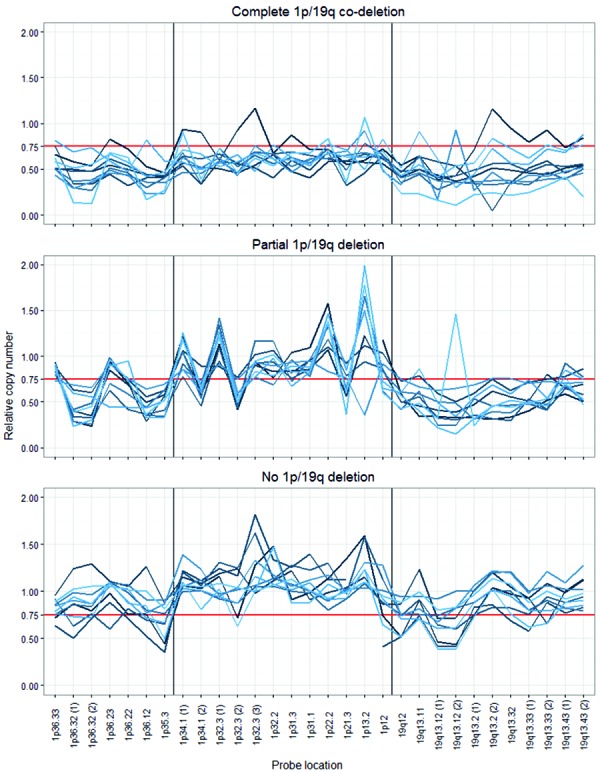
Line plots of MLPA data for each tumor grouped according to the proposed criteria. Thick red horizontal line indicates the threshold of 0.75.

**Figure 4.**
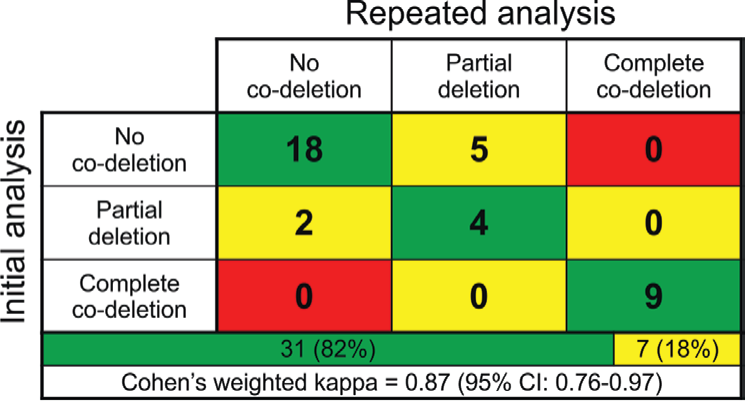
Confrontation of the results of both analyses (initial analysis in rows, repeated analysis in columns). Concordance was marked as green, partial discordance as yellow and discordance as red. Bottom rows show the proportions between concordant and partially discordant cases and κ-statistics. Cohen’s weighted κ-statistics was calculated using the order as in the table: no-codeletion (0), partial co-deletion (1), complete co-deletion (2).

There is no consensus on scoring criteria for 1p/19q MLPA and there have been no attempts at their clinical validation (probe locations presented in Figure 1). Several authors have proposed different criteria [23, 24, 25], but none of them aimed at recognizing the segmental deletions [26]. Therefore, we have decided to design a simple and straightforward approach clearly separating the complete co-deletion from the isolated segmental deletions and normal copy numbers. For this purpose we applied the commonly used threshold of 0.75 and considered the commonly affected subtelomeric 1p region (1p35-36) separately from the other part of the chromosome. A complete co-deletion is called if the majority of loci within each region (subtelomeric 1p, rest of 1p, 19q) is deleted (< 0.75), while isolated segmental deletion refers to the subtelomeric 1p (but not the rest of 1p) significantly affected (exemplary results are presented in Figure 2). Thus, we recognized co-deletions in 36 cases (24%), isolated segmental deletions in 48 cases (32%) and no deletion in 66 cases (44%; the association with other tumor features is depicted in Figure 3). To assess the reproducibility we reanalyzed a set of 38 randomly selected cases. We observed full concordance in discrimination between co-deletion and lack of co-deletion (κ = 1) and high concordance when isolated segmental deletions were regarded separately (κ = 0.87, Figure 4). Finally, we investigated how the (clinically significant) distinction between co-deletion and lack thereof according to different criteria (ours and the ones by Natté and Jeuken [23, 24]) aligned with the observed histology and other markers (Table 4). The criteria by Jeuken et al. [24] are clearly the most specific, but have a low sensitivity. In contrast, the criteria by Natté et al. [23] are more sensitive, but in turn the specificity is largely reduced. Our approach conferred similar sensitivity to Natté’s criteria, but without the unnecessary specificity costs (Table 4). 

## Discussion 

Traditionally, diffusely infiltrating gliomas have been classified according to the morphology of the dominant population of tumor cells as oligodendrogliomas, astrocytomas, or oligoastrocytomas in case of a balanced distribution of tumor components [16], while the grade is attributed according to mitotic activity, cellularity, and nuclear atypia along with the characteristic features of malignancy (necrotic foci and microvascular proliferations). The aspect of interobserver agreement has been criticized on numerous occasions [8]. In this case, the problems with reproducibility primarily result from the fact that discrimination of diffusely infiltrating gliomas is a somewhat arbitrary division of a two-dimensional spectrum (astrocytic/oligodendroglial and low/high grade) into a set of specific entities (extensively investigated by Venteicher et al. [27]). Still, such a distinction is clinically useful and critically affects patient management [28]. Certain molecular profiles are also postulated to carry a valuable prognostic information and might be applied to identify cases requiring intensified treatment (e.g., gliomas especially prone to progression) [9]. With the recent 2016 update of WHO Classification of Tumors of the Central Nervous System molecular markers have become an integral part of diagnostic brain tumor typing [1]. However, the issue of selecting the analytical methods and defining diagnostic criteria is left open with no specific recommendations [1, 28]. For these reasons, we aimed to evaluate the usefulness and reliability of a combined set of widely available methods for routine diagnostics in the real-life setting. 

*TERT* promoter mutation analysis is virtually limited to DNA-based methods, sequencing and pyrosequencing in particular, however, no round-robin trial assessing their analytic performance has been published. Due to the high G-C content in the *TERT* promoter sequence, some next-generation sequencing (NGS) trials reported difficulties in the assessment of *TERT* promoter sequence [29, 30]. In contrast, direct sequencing allows the specific optimization of PCR conditions for better sensitivity and specificity. The anti-IDH1^R132H^ antibody is well known for its sensitivity and specificity [1], which we can confirm in our observations. Despite some variability between different batches of the antibody, it is clearly the optimal method to identify this alteration. To detect other mutations we employed both direct sequencing and MLPA. The former appeared to be more specific with some sensitivity issues (especially in cases with low tumor cell content), which could often be solved by sequencing of the nested PCR products. On the other hand, MLPA presented a better sensitivity, yet, with occasional non-specific peaks. Thus, neither technique appears to be significantly superior, but their integration offers the most reliable results. At single-case level, repetition of a doubtful result is also worth considering. In our study cohort we observed a lower frequency of *IDH* mutations than expected; however, it may result from the inclusion of all consecutive diffuse glioma cases undergoing routine diagnostics. 

In gliomas, 1p/19q co-deletion is the most difficult biomarker from the perspective of routine diagnostics. Firstly, there are numerous alternative methods, among which only the cell-based ones (FISH, CISH) have clearly defined criteria and only FISH was clinically validated [14]. However, FISH and CISH are expensive and time-consuming techniques, which is a certain issue for routine diagnostics. Secondly, apart from the combined loss of whole arms 1p and 19q due to the unbalanced translocation [31], other copy number alterations affecting these regions may be detected. Isolated segmental deletions within 1p35-36 and 19q are a relatively common finding in astrocytic tumors [26, 32, 33]. In such cases, the major part of 1p is intact and it is clear that these alterations result from a different mechanism than the complete co-deletion. Their clinical significance has not been elucidated yet; however, these isolated deletions should be clearly distinguished from the complete co-deletion. The commonly used FISH probes are mapped to 1p36 and 19q13, thus making this method prone to error of confusing the isolated segmental deletions (present in astrocytoma) with the complete co-deletion (present in oligodendroglioma) [32]. Note that the clinical trial validating FISH was conducted on a selected group of tumors with anaplastic oligodendroglial histology, thus, avoiding the cases with potential isolated sequential deletions [14]. These problems may easily be overcome by using additional or differently located FISH probes, but such a tool would have to be validated from the start. 

On the other hand, the DNA-based methods are not perfectly suited for routine work-up either. Firstly, these techniques are highly dependent on the quality of isolated nucleic acids, which is poor in the commonly used FFPE blocks, as well as on an adequate tumor cell content, which may be low if only a small biopsy from the infiltration zone is available. Secondly, these methods currently have no clearly defined scoring criteria, while clinical validation is even more distant. Nevertheless, among these techniques aCGH and snpArrays are probably the most reliable methods, however, their costs largely exceed the values acceptable for routine use. Simple loss of heterozygosity (LOH) analysis provides only limited information per single analysis and depends on heterozygous sites (thus, requires mapping of numerous loci). MLPA presents as a promising method as it allows to quantitatively analyze numerous loci throughout the chromosomal arms at relatively low costs. So far, this technique has been reported in the literature to identify 1p/19q status in gliomas for 6 times [23, 24, 25, 34, 35, 36]. In three papers, analyses were performed on DNA isolated from FFPE blocks [23, 24, 25]. Therefore, we aimed to investigate the optimal scoring criteria for 1p/19q MLPA and to assess its routine applicability. 

Our biggest challenge was to design clinically meaningful scoring criteria, which would still be simple and easy to apply. The probe set is mapped to 19 1p loci and 11 19q loci, thus, it has to be defined when to consider a locus deleted (threshold) and when to consider a region deleted (number of deleted loci). The manufacturer recommendations (threshold at 0.65) have been developed for relatively homogeneous samples (e.g., cell cultures) and seem too strict for routine diagnostics (heterogeneous FFPE samples). In the literature, only 3 authors applied MLPA for 1p/19q in FFPE samples. Two of these groups employed a threshold of 0.75 [23, 25] and the third – of 0.80 [24], however, none considered the 1p36 partial deletions. Our goal was to clearly distinguish the clinically significant co-deletion from the potentially misleading isolated segmental deletions and from the normal status. Therefore, we employed the threshold of 0.75 and considered separately the subtelomeric (1p35-36) and remaining part (paracentromeric) of 1p. Obviously, the selection of the sample with highest tumor cell content is crucial for this analysis. However, if in a given case only a sample with significant addition of normal cells is available, one might consider to shift the threshold to 0.80 or to refrain from performing the analysis. 

Subsequently, we investigated the overall performance of our MLPA cutoff criteria in association with the features typical for oligodendrogliomas (oligodendroglial histology, *IDH* and *TERT* mutations and no ATRX loss). For comparison, we performed a similar analysis for the two previously published MLPA criteria [23,24]. When doing so, we were not able to address the criteria by Trabelsi et al. as they were not stated by the authors [25]. The poorer performance of Jeuken’s criteria may be explained by their excessive strictness [24]. On the other hand, several clearly astrocytic tumors (with purely astrocytic morphology, ATRX loss and lack of *TERT* promoter mutation) were identified as 1p/19q co-deleted using Natté’s criteria [23]. The problem with their specificity apparently resulted from the very high correlation of the copy number values for 1p35-36 and 19q12-13 loci (Spearman’s rho ranging from 0.247 to 0.867), so that some cases with isolated segmental deletion were marked as co-deleted. In contrast, our approach seems to offer the optimal balance between sensitivity and specificity. It is also adequate in terms of reproducibility as we could achieve full concordance in distinguishing the clinically relevant co-deletion from its lack. The separate distinction of isolated segmental deletions decreased the overall reproducibility, but it remained within a satisfactory range (κ = 0.87). Of note, in MLPA analysis of non-tumorous CNS specimens obtained from epilepsy surgery, we observed deletions of a limited number of DNA loci on both chromosome arms (1p and 19q; data not shown). This seems to be an artifact of formalin-fixed and paraffin-embedded neurosurgical tissue specimens, which may be dependent on the duration of formalin fixation. The number of deleted loci is, however, clearly below the proposed MLPA cutoff criteria of 1p/19q co-deletion status. 

One of the limitations of our study is the lack of catamnesis, however, the study was intended to evaluate the applicability of combined immunohistochemistry, direct sequencing, and MLPA analysis for molecular diagnostic testing of IDH, 1p/19q, and *TERT* status and not the prognostic value of these markers, which would require an adequately designed prospective study. Secondly, formalin fixation of the tissue may also affect the analytical performance of these methods, which should give better results with frozen tissue or formalin-free fixatives; however, we aimed to investigate the usefulness of the methods in a real-life diagnostic setting, where such materials are rarely available. Additionally, the fact that the cases in the present series were categorized according to the 2007 Classification [16] instead of the current one (2016) [1] could be regarded as a limitation. However, the purpose of our study was to assess the applicability of molecular testing of diffuse gliomas using tumors in which molecular testing has not been done before. Therefore, we retained the original histological diagnoses of the cases included in this study. 

In summary, we tested under real-life conditions in a consecutive series of 165 diffuse gliomas the practicability of combined immunohistochemistry, direct sequencing, and MLPA analysis for assessment of the most important molecular markers relevant for diagnostic tumor typing (practical aspects of each method are summarized in Table 5). Our data confirm that immunostaining is a straightforward way to detect IDH1^R132H^ mutation, while other IDH mutations are optimally identified by integration of direct sequencing and MLPA analysis; *TERT* promoter sequencing seems to be useful for the detection of mutations, but its analytical performance (sensitivity, specificity) remains to be tested. In our hands, MLPA is a reliable and reproducible method to identify 1p/19q co-deletion and we believe that this approach can be recommended for routine diagnostic work-up in other laboratories. 

## Acknowledgment 

We would like to thank Professor Harald Heinzl, Center for Medical Statistics, Informatics, and Intelligent Systems, Medical University of Vienna, for his constructive criticism and helpful comments. We are grateful to Michaela Kreiter for her technical support. 

## Funding 

Michał Bieńkowski was supported by the Healthy Ageing Research Centre project (REGPOT-2012-2013-1, 7FP). 

## Conflict of interest 

The authors declare no conflict of interests. 

**Table 1.** PCR conditions and primer sequences.

PCR conditions			
Initial denaturation	95 °C/15 min		
10 cycles	94 °C/30 s	60 °C/30 s	72 °C/90 s
		(0.5 °C decrement each cycle)	
25 cycles	94 °C/30 s	55 °C/30 s	72 °C/90 s
Final elongation	72 °C/6 min		
Primer sequences			
TERTProm1-MTR	5’-CAGGAAACAGCTATGACGCACAGACGCCCAGGACCGCGCT		
TERTProm1-M13	5’-GTAAAACGACGGCCAGTTTCCCACGTGCGCAGCAGGACGCA		
IDH1/Ex4P-MTR (nested)	5’-CAGGAAACAGCTATGACTGGGTGGCACGGTCTTCAG		
IDH1/Ex4P-M13 (nested)	5’-GTAAAACGACGGCCAGTCAAAATCACATTATTGCCAACATGAC		
IDH1/Ex4-MTR	5’-CAGGAAACAGCTATGACTCACTCCTGATGAGAAGAGGGTTG		
IDH1/Ex4-M13	5’-GTAAAACGACGGCCAGTAAAATGTGTTGAGATGGACGCC		
IDH2/Ex4-MTR	5’-CAGGAAACAGCTATGACGCTGTGTTGTTGCTTGGGGTTC		
IDH2/Ex4-M13	5’-GTAAAACGACGGCCAGTGGGTGAAGACCATTTTGAAAGTGC		

**Table 2.** Study group summary.

	N	M/F	Mean age
Astrocytic tumors	80	31/39	49.1
– Astrocytoma	31	12/19	42.3
– Anaplastic astrocytoma	49	29/20	53.5
Oligoastrocytic tumors	54	32/22	42.3
– Oligoastrocytoma	26	12/14	37.4
– Anaplastic oligoastrocytoma	28	20/8	46.6
Oligodendroglial tumors	31	11/20	43.1
– Oligodendroglioma	18	7/11	40.7
– Anaplastic oligodendroglioma	13	4/9	46.4

**Table 3.** Distribution of markers (*TERT* and IDH mutations, 1p/19q complete co-deletion, ATRX loss, TP53 and EGFR expression) for each diagnosis. 1p/19q status according to the criteria discussed throughout the manuscript.

	IDH mutation		*TERT* mutation		Compl. 1p/19q co-del		ATRX loss		TP53 expression		EGFR expression	
Astrocytic tumors	21/80	26%	37/72	51%	1/68	1%	24/78	31%	38/80	48%	47/77	61%
– Astrocytoma	13/31	42%	15/29	52%	0/27	0%	14/30	47%	11/31	35%	18/29	62%
– Anaplastic astrocytoma	8/49	16%	22/43	51%	1/41	2%	10/48	21%	27/49	55%	29/48	60%
Oligoastrocytic tumors	46/54	85%	37/54	69%	17/53	32%	25/53	47%	30/53	57%	34/53	64%
– Oligoastrocytoma	22/26	85%	17/26	65%	9/26	35%	12/26	46%	13/26	50%	16/26	62%
– Anaplastic oligoastrocytoma	24/28	86%	20/28	71%	8/27	30%	13/27	48%	17/27	63%	18/27	67%
Oligodendroglial tumors	24/31	77%	22/31	71%	18/29	62%	3/31	10%	9/31	29%	17/31	55%
– Oligodendroglioma	14/18	78%	13/18	72%	10/16	63%	1/18	6%	4/18	22%	9/18	50%
– Anaplastic oligodendroglioma	10/13	77%	9/13	69%	8/13	62%	2/13	15%	5/13	38%	8/13	62%

**Table 4.** Comparison of tumors with and without the 1p/19q co-deletion identified according to different criteria. The p-values were calculated using Fisher’s exact test or χ^2^-test depending on sample size.

			Co-deletion	No co-deletion	p-value
Jeuken et al. [24]	Histology	Astrocytic	0	68	0.0002
		Mixed	7	46	
		Oligodendroglial	6	23	
	ATRX	Loss	0	49	0.0051
		Retention	13	87	
	*TERT*	Mutant	12	78	0.0138
		Wild-type	1	58	
	IDH	Mutant	13	74	0.0013
		Wild-type	0	63	
Natté et al. [23]	Histology	Astrocytic	4	64	< 0.0001
		Mixed	24	29	
		Oligodendroglial	18	11	
	ATRX	Loss	6	43	0.0006
		Retention	40	60	
	*TERT*	Mutant	42	48	< 0.0001
		Wild-type	4	55	
	IDH	Mutant	37	50	0.0002
		Wild-type	9	54	
Ours	Histology	Astrocytic	1	67	< 0.0001
		Mixed	17	36	
		Oligodendroglial	18	11	
	ATRX	Loss	1	48	< 0.0001
		Retention	35	65	
	*TERT*	Mutant	34	56	< 0.0001
		Wild-type	2	57	
	IDH	Mutant	31	56	< 0.0001
		Wild-type	5	58	

Definitions of 1p/19q co-deletion criteria: according to Jeuken et al. [24]: complete co-deletion if all loci ≤ 0.8. according to Natté et al. [23]: complete co-deletion if > 50% of loci on both chromosomes ≤ 0.75. Ours: complete co-deletion if the majority of loci within each of the regions: 19q, subtelomeric 1p (1p35-1pter) and pericentromeric 1p ≤ 0.75.

**Table 5.** Strengths and weaknesses of the different methods used for *IDH1*, *IDH2*, and *TERT* mutation testing, and for 1p/19q deletion testing.

Strengths and weaknesses of the different methods	
Immunohistochemistry (IHC)	IHC detects sensitively and specifically the IDH^R132H^ mutation, but no other IDH1 mutations and no IDH2 mutations
Direct DNA sequencing	Direct DNA sequencing detects specifically all IDH1, IDH2, and TERT mutations. However, sensitivity of mutation detection is limited because of the admixture of non-mutated DNA from normal cells
Next-generation sequencing (NGS)	The detection of *IDH1*, *IDH2*, and *TERT* promoter mutations by means NGS may be less sensitive as compared to single gene sequencing for the following reasons: 1) NGS uses less DNA and/or a lower number of PCR-cycles 2) It is much easier to optimize PCR condition for an individual set of primers as compared to a large panel
Multiplex-dependent probe amplification (MLPA)	MLPA allows for simultaneous detection of DNA losses of a large number of chromosomal loci, with reliable detection of DNA deletions if the right cutoff criteria are carefully elaborated. Furthermore, MLPA allows also for a highly sensitive detection of single amino acid mutations, such as the *IDH1* *^R132H^* mutation. A drawback is, however, that the currently available MLPA probe set does not cover all *IDH1* and *IDH2* mutations, and also not the *TERT* mutations
Fluorescent in situ hybridization (FISH) and chromogenic in situ hybridization (CISH)	Both techniques detect deletions of large chromosomal fragments at the single cell level, however, these methods are more technically challenging and their scoring is more time-consuming. In addition, with the currently employed probes it is possible to confuse a partial 1p loss with the complete 1p/19q co-deletion.
